# Efficacy of Several Plants Extracts on the Tunneling Activity and Survival of Subterranean Termites (*Coptotermes gestroi* and *Globitermes sulphureus*)

**DOI:** 10.21315/tlsr2019.30.1.3

**Published:** 2019-01-31

**Authors:** Noor Hazwani Bakaruddin, Abdul Hafiz Ab Majid

**Affiliations:** Household and Structural Urban Entomology Laboratory, Vector Control Research Unit, School of Biological Sciences, Universiti Sains Malaysia, 11800 USM Pulau Pinang, Malaysia

**Keywords:** Plant Extracts, Tunneling Activity, *Coptotermes gestroi*, *Globitermes sulphureus*, Ekstrak Pokok, Aktiviti Menerowong, *Coptotermes gestroi*, *Globitermes sulphureus*

## Abstract

This study examined the tunneling activity and the behaviour of two subterranean termites, *Globitermes sulphurues* and *Coptotermes gestroi* treated with four plant extracts, namely *Pyllanthus niruri, Azadirachta indica, Leucaena leucocephala* and *Andrographis paniculata*. All plants were extracted with three different solvents (methanol, hexane and water) and were diluted into three concentrations (500, 5,000 and 10,000 ppm). A group of 50 workers and 2 soldiers were tested and observed daily during the exposure in order to determine their survivorship and behaviour. Both sections were scored to determine their tunneling activities after seven days. There was a significant difference of tunneling activities of *C. gestroi* on sand treated with plant extracts (X^2^(2) = 31.790, *p* < 0.0001) with a mean rank of 8.50 for methanolic extracts and 32.50 for both hexane and water extracts. Meanwhile, no significant difference was observed on tunneling activity of *G. sulphureus* on treated sand (X^2^(2) = 2.200, *p* = 0.333) with a mean rank of 20.72 for methanolic extracts, 26.31 for water extracts and 26.47 for hexane extracts. Results showed that plants extracted with methanol demonstrated strong repellent properties with 0 tunneling activity on the treated sand and low survivorship of both termites. Moreover, both termites did not survive (0%) when they were treated with methanolic extracts at 10,000 ppm. They also displayed a different behaviour post-exposure such as avoidance, gradually losing the ability to walk and low feeding consumption. These results indicate that there is a strong termiticidal activity of plants extracted with methanol against *C. gestroi* and *G. sulphureus*.

## Abstract (ms)

Kajian ini mengkaji aktiviti menerowong dan tingkah laku anai-anai bawah tanah, *Globitermes sulphurues* dan *Coptotermes gestroi* apabila dirawat dengan empat ekstrak tumbuhan iaitu *Pyllanthus niruri*, *Azadirachta indica*, *Leucaena leucocephala* dan *Andrographis paniculata* melalui kaedah piring petri. Tumbuh-tumbuhan telah diekstrak dengan pelarut yang berbeza (metanol, heksana dan air), dan mempunyai tiga kepekatan (500, 5,000 dan 10,000 ppm). Seramai 50 ekor pekerja dan 2 ekor askar anai-anai telah digunakan untuk ujikaji dan setiap hari, mereka diperhatikan sepanjang ujian dijalankan untuk menilai kemandirian anai-anai dan tingkah laku mereka. Selepas tujuh hari, keduadua bahagian telah dinilai untuk menentukan aktiviti terowong mereka. Ujian Kruskal-Walis H menunjukkan bahawa aktiviti menerowong daripada *C. gestroi* adalah berbeza antara satu sama lain apabila dirawat dengan tumbuhan yang telah diekstrak oleh pelarut yang berbeza (X^2^ (2) = 31.790, *p* < 0.0001), dengan pangkat min 8.50 untuk ekstrak tumbuhan metanol dan 32.50 untuk ekstrak tumbuhan heksana dan air. Sementara itu, *G. sulphureus* tidak menunjukkan perbezaan yang ketara dalam aktiviti terowong pada pasir terawat, (X^2^ (2) = 2.200, *p* = 0.333) dengan pangkat purata 20.72 untuk ekstrak tumbuhan metanol, 26.31 untuk ekstrak tumbuhan air dan 26.47 untuk ekstrak tumbuhan heksana. Hasil kajian menunjukkan bahawa semua tumbuhan yang diekstrak dengan metanol menunjukkan ciriciri mengusir yang kuat dengan 0 aktiviti terowong pada pasir terawat dan kelangsungan anai-anai yang rendah. Selain itu, 0% kelangsungan telah dilihat apabila kedua-dua spesis anai-anai dirawat dengan ekstrak tumbuhan metanol pada 10,000 ppm. Kedua-dua spesis anai-anai ini menunjukkan tingkah laku yang berbeza selepas tamat ujikaji seperti kelakuan mengelak, kehilangan keupayaan untuk berjalan secara perlahan dan makan dalam kuantiti yang sedikit. Hasil kajian ini berjaya membuktikan bahawa tumbuhan yang diekstrak dengan metanol mempunyai aktiviti racun anai-anai yang tinggi terhadap *C. gestroi* dan *G. sulphureus*.

## INTRODUCTION

Foragers of subterranean termites are known to search for foods by tunneling through soil (Cao & Su 2014). Generally, they construct a tunnel and gallery systems underneath and above the soil (Gautam & Henderson 2012). The efficiency of food finding by subterranean termites depends on the organisation of the tunnel systems (Traniello & Robson 1995). In addition, foraging behaviour is influenced by numerous factors such as humidity, soil texture, moisture availability, soil compaction and preformed tunnel cavities (Cornelius & Osbrink 2010; Ku *et al*. 2013). Due to their behaviour, subterranean termites able to reach and attack buildings that initiated from a nest in the ground (Kuswanto *et al*. 2015). Subterranean termites such as *Coptotermes formosanus* is known for their aggressive feeding behaviour, hence, they are considered as one of the important pests in Japan, Taiwan and China (Ngee *et al*. 2004).

Controls of subterranean termite are crucial in order to protect any building structure and its component. One of the control measures includes a soil barrier treatment practically used as one of the main strategies to protect building structures from subterranean termites (Rust & Saran 2006). The treatment is employed basically by creating a chemical barrier to prevent termite attack (Bläske *et al*. 2003). According to Su *et al*. (1982), insecticides can be categorised as repellent, toxic, and non-repellent, or non-repellent with delayed toxicity. The intensive use of pesticides produces side effects on many beneficial insects and show acute and chronic effects on living organisms (Abudulai *et al*. 2001). The use of chemical insecticides for soil and crop treatments has been continuously allowed for the present time due to the lack of any effective substitute (Alshehry *et al*. 2014).

Recently, there has been an increasing interest for the development of environmentally friendly products such as botanical pesticides, microbial sprays and insect growth regulators (Bläske & Hertel 2001). Several studies have been carried out to evaluate the effect of natural products. Manzoor *et al*. (2011), for instance, reported that *Curcuma longa* extract was found to be more efficient in soil treatments to protect food substrate against *Heterotermes indicola* as termites did not cross the barrier. In addition, leaf and seed extracts of *Jatropha curcas*, *Annona* crude seed extract, rhizome of *Curcuma longa* extract, leaves of *Nerium indicumi* extracts and bark of *Melia azedarach* extracts are among plants which were investigated to evaluate their effects on tunneling activities of subterranean termites (Nisar *et al*. 2012; Acda 2014).

A lower termite, *C. gestroi* is notoriously known as an important pest species as it has caused numerous structural damages worldwide (Li *et al*. 2009). This species attacks buildings in urban, suburban or rural areas (Kirton & Azmi 2005). Meanwhile, *G. sulphureus* is a well-known secondary pest (Neoh *et al*. 2011), commonly found in premises that were treated with bait following the previous infested termites (Lee *et al*. 2007). This higher termite species attacks agricultural crops such as rubber, coconut, oil palm and sugar cane plantations (Tho 1992). This species can occasionally be found in building structures in rural and suburban areas (Kirton & Azmi 2005). In urban areas, both species have become a major problem by causing negative economic effects, devaluing property, damaging crops and necessitating household repair. Besides, severe damages caused by these species could occur in a relatively short time (Scheffrahn & Su 2008).

In this study, four plants were selected based on their chemical compounds, as well as their advantageous properties such as insecticidal activity, repellency to pests, deterrent to feeding, insect growth regulation and toxicity to insect pests. For example, methanolic and ethyl acetate extracts of *A. paniculata* was reported to be highly effective in controlling weevil, *Callosobruchus chinensis* L (Bright *et al*. 2001). In a study by Elango *et al*. (2012), the ethyl acetate extract exhibited the highest termite mortality compared to others. Meanwhile, *Phyllanthus niruri* composes of a series of chemical compounds such as tannins, lignans, coumarins, flavonoids, terpenes, alkaloids, phenylpropanoids and saponins that widely distributed in stems, leaves and roots (Bagalkotkar *et al*. 2006). Adding to this, *A. indica* contains limonoid, a compound with antifeeding activity, growth inhibition, oviposition, contact poison, and repellent activity beneficial in insects and mites control management (Himmi *et al*. 2013; Schutter *et al*. 1995). *Leucaena leucocephala*, on the other hand, was previously reported as a mimosine producer that involved in mosquito larval mortality (Aarthi *et al*. 2011). Owing these insecticidal values, the purpose of this study was hereby to determine the efficacy of crude extracts derived from *L. leucocephala, A. paniculata, A. indica and P. niruri* on the tunneling activity and survivorship of two main building pests in Malaysia, namely *C. gestroi* and *G. sulphureus*.

## MATERIALS AND METHODS

### Termite Collection

One colony of *C. gestroi* and four colonies of *G. sulphureus* were collected in Universiti Sains Malaysia (USM) campus and Teluk Bahang, Pulau Pinang. Termites were brought to the Household and Structural Urban Entomology Laboratory, School of Biological Sciences, USM for further analysis. They were placed in a plastic container (covered with black plastic) supplied with soil and wood as food sources and were kept in the dark. They were reared at room temperature (28 ± 2°C) with a relative humidity of 70% ± 10). Prior to the experiment, termites were previously separated from debris using the bridging method by allowing them to access five stacks of pre-wetted pine blocks (20 × 10 cm) (Tamashiro *et al*. 1973). Then, the termites were counted and transferred to a plastic Petri dish (90 mm × 15 mm, Ideal Healthcare, Malaysia) lined with moistened filter paper (90 mm, Advantec, Japan).

### Plant Collection

The dried form of four plant samples namely, *Andrographis paniculata, Leucaena leucocephala*, *Azadirachta indica* and *Pyllanthus niruri* were acquired from Herbagus Sdn. Bhd. Only the leaf part was used for the plant crude extraction, except for *P. niruri*, where the whole plant parts were used for the same purpose.

### Soxhlet Extraction

Each dried plant sample (30 g) was loaded into the thimble. The round bottom flask was filled with 150 ml of methanol (99.5%, EMSURE, Germany) or hexane (96.0%, EMSURE, Germany) as a solvent and was heated using an isomantle heater. The solvent was evaporated through the Soxhlet apparatus. This extraction procedure was continuously repeated for approximately 20–30 cycles for 4 to 6 h at 110°C–130°C, until the entire solvent evaporated and condensed. The remaining solution was continually evaporated by using a rotary evaporator to obtain a concentrated extract. The concentrated extract was then evaporated in the oven at 80°C for 48 h and stored in the freezer at 4°C for further analysis.

### Water Extracts by Maceration Extraction

Each dried plant sample (30 g) was extracted with distilled water (180 mL) for 24 h. At first, the plant material was mixed with distilled water and was frequently shaken during the first six hours and then was allowed to stand for another 18 h. Next, the extract was filtered through a Whatman filter paper and concentrated by using a rotary evaporator. The obtained extracts were kept at 4°C until used.

### Preparation of Plant Extract Solutions and Concentrations

The plant extracts were prepared in concentrations of 500, 5,000 and 10,000 ppm for each plant extract. A stock solution (10,000 ppm) was prepared by diluting 3 g of the crude extract in 300 mL of respective solvents. The lower concentrations were subsequently prepared from this stock solution.

### Preparation of Treated and Untreated Sands

The treated sand was prepared by mixing 30 g of sand with 3 mL of plant extract solution of three different concentrations (500, 5,000, 10,000 ppm). For untreated/solvent-treated sand, 30 g of sand were mixed with three blank solvents (methanol, hexane, water). All treated sands were kept for 24 h in a fume hood at a room temperature to allow solvent evaporation.

### Tunneling Bioassay

The bioassay method was according to Yeoh and Lee (2007) with slight modifications. Petri dishes (90 mm × 15 mm, Ideal Healthcare, Malaysia) were divided into two sections and were used as a test arena. The first section consisted of 30 g of sand treated with plant extract solutions, while the other section was filled with 30 g of sand treated with blank solvents. Each plant extract with different concentrations was prepared in three replicates. A Petri dish containing 30 g of solvent-treated sand in one section and 30 g of blank sand in the other section was used as a control. A pre-weight filter paper (1.5 cm diameter) was placed in each section and was served as a food. Fifty workers and two soldiers of both termite species were introduced in the middle of the Petri dishes and were allowed to move and tunnel freely. All Petri dishes were placed in an incubator and maintained in darkness at 26 ± 2°C and 65 ± 5% relative humidity.

The tunneling activity was observed daily and the number of surviving termites was recorded after seven days. After seven days, both sections were scored with 0 for no tunneling activity, 1 for tunneling activity covering ≤25% of total arena, 2 for tunneling activity covering 26%–50% of total arena, 3 for tunneling activity covering 51%–75% of total arena, and 4 for tunneling activity covering ≥75% of the total arena. The weight of the filter paper after the treatment was recorded to estimate the feeding consumption rate.

### Statistical Analysis

Statistical analysis was performed IBM SPSS Statistics Version 22. Data of tunneling activity in treated and untreated sections were analysed with Kruskal-Wallis (KW) analysis of variance (ANOVA) for each plant extract solution. The Mann–Whitney U test was used to compare mean of tunneling activity between two concentration groups of each plant solution. The one-way ANOVAs was performed and the Tukey’s test at *P* < 0.05 was done to assess the significant differences between the concentrations of each plant extract in order to determine the termite survivorship and feeding consumption.

## RESULTS

### Tunneling Activity and Survivorship of *C. gestroi* and *G. sulphureus*

**Figure 1:**
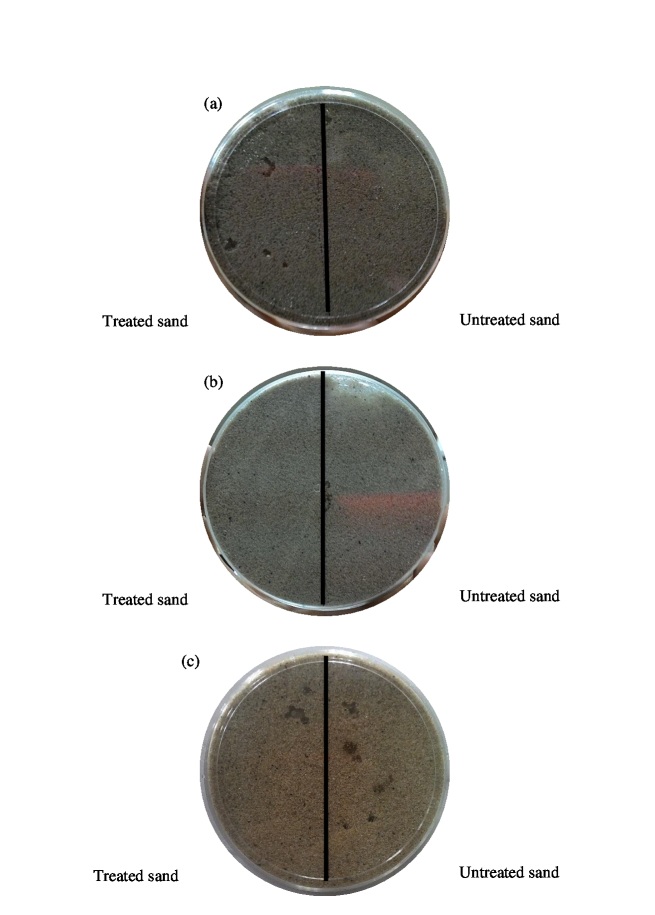
Tunneling activity of *G. sulphureus* in Petri dishes containing untreated and treated sands: (a) methanol treated, (b) hexane treated and (c) control (water).

**Figure 2:**
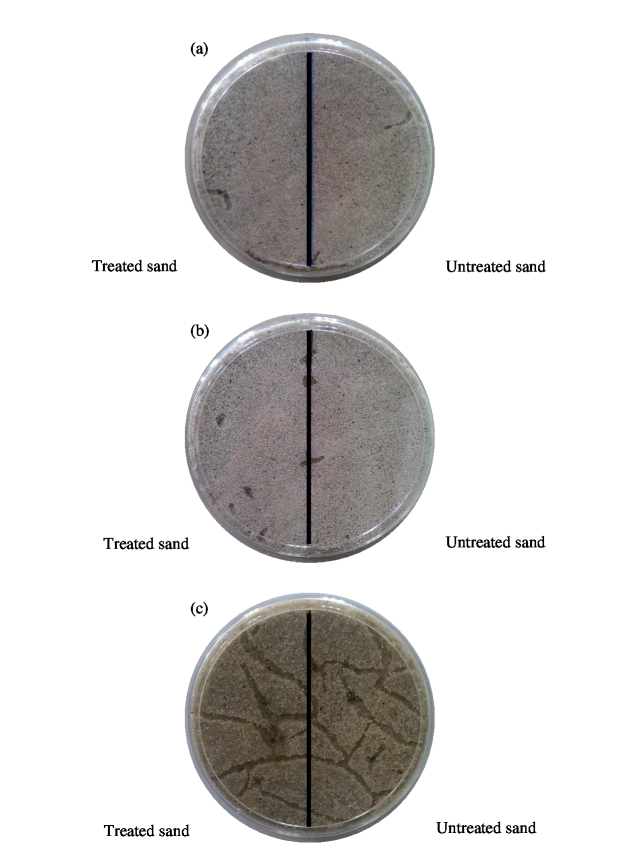
Tunneling activity of *C. gestroi* in Petri dishes containing untreated and treated sands: (a) methanol treated, (b) hexane treated and (c) control (water).

Plant-derived extracts used in this study exhibited both repellent and non-repellent activities towards the termite species tested among the extracts obtained from different plants. There was no significant difference in tunneling activity in treated sections between different plant extracts against *C. gestroi*, X^2^(3) = 1.413, *p* = 0.702. In addition, Kruskal-Walis H test exhibited a significant difference of tunneling activity on sand treated with plants extracted with different solvents on *C. gestroi*, X^2^(2) = 31.790, *p* < 0.0001, with a mean rank of 8.50 for methanolic plant extracts and 32.50 for hexane and water extracts. Meanwhile, *G. sulphureus* showed no significant difference on tunneling activity on treated sands, X^2^(2) = 2.200, *p* = 0.333 with a mean rank of 20.72 for methanolic extracts, 26.31 for water extracts and 26.47 for hexane extracts. A Kruskal-Wallis H test showed that there was a significant difference in tunneling activity in the treated sections between different plant extracts against *G. sulphureus* (X^2^(3) = 11.115, *p* = 0.011) with a mean rank treated section score of 33.43 for *P. niruri*, 74.83 for *L. leucocephala*, 65.32 for *A. paniculata* and 63.42 for *A. indica*. In controls, tunneling activity in both treated and untreated sections were constant and uninterrupted with high number of survivors in the Petri dish (Figs. 1–2).

**Figure 3:**
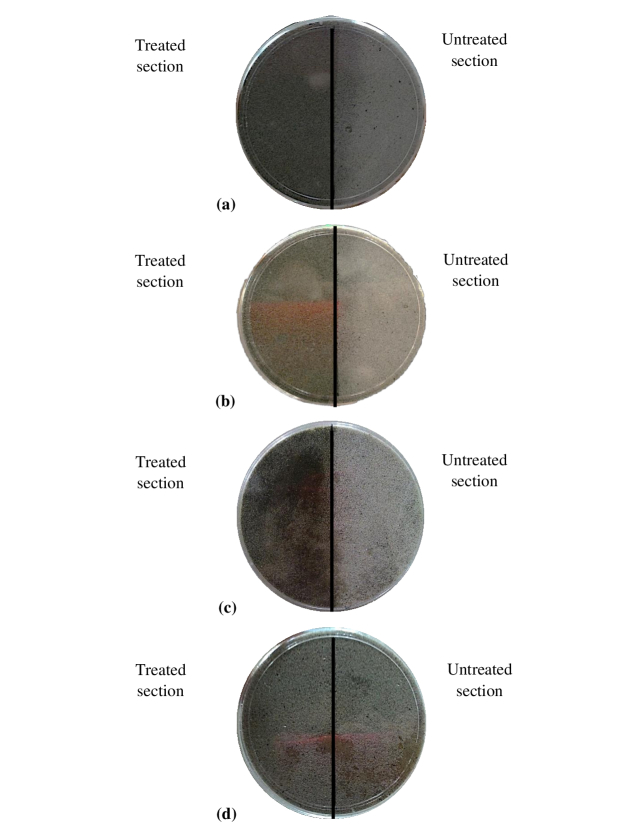
Tunneling activity of *G. sulphureus* on methanol plant extracts at 10,000 ppm (a) *P. niruri*, (b) *A. paniculata*, (c) *A. indica* and (d) *L. leucocephala* after *seven* days of exposure.

**Figure 4:**
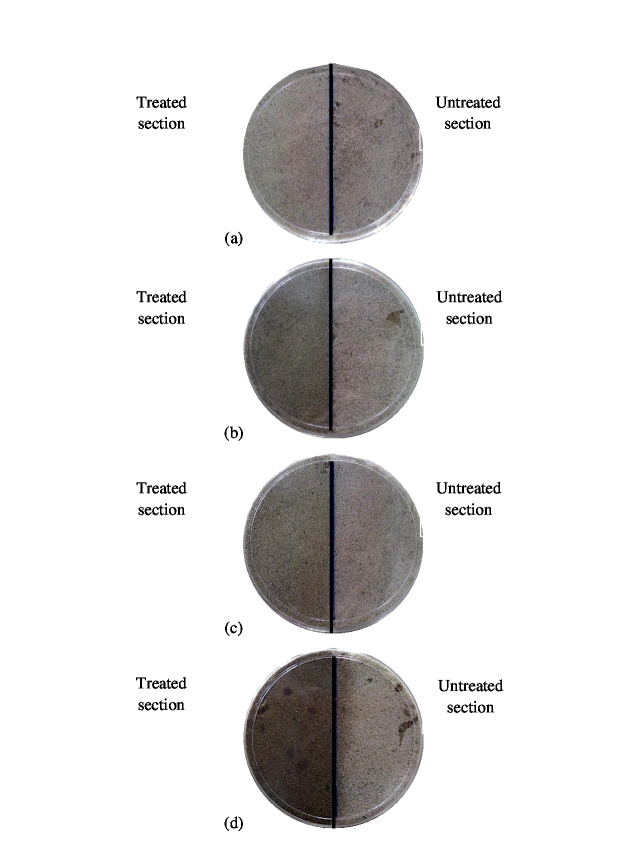
Tunneling activity of *C. gestroi* on methanol plant extracts at 10,000 ppm (a) *P. niruri*, (b) *A. paniculata*, (c) *A. indica* and (d) *L. leucocephala* after seven days of exposure.

Effects of methanolic extracts on tunneling activity and survivorship of *C. gestroi* and *G. sulphureus* are summarised in Table 1 and Table 2. Methanolic extracts showed high repellent activity where low tunneling activity was observed in all treated sections particularly in sections treated with a concentration of 10,000 ppm (Figs. 3 and 4). A Kruskal-Wallis H test showed that there was no a statistical significant difference of tunneling activity in treated sections between different of methanolic extracts against *C. gestroi*, X^2^(3) = 2.195, *p* = 0.533 and *G. sulphureus*, X^2^(3) = 5.262, *p* = 0.154. In methanolic extract, *G. sulphureus* was found to walk freely on the surface of two sections at all concentrations used until Day 2. After approximately three days, termites stopped any feeding activity on both sections and their walking ability slowly reduced. Once the termites started dying, the living termites in the arenas shifted to the untreated section and stayed on the surface of the untreated section until day-7 and slowly became immobile except at 500 ppm. No tunneling activity by *G. sulphureus* was observed at the treated section at seven days exposure at all concentrations, *P. niruri* caused 100% mortality of *G. sulphureus* at all concentrations, whereas, *L. leucocephala* and *A. paniculata* caused 100% mortality in sections treated with 5,000 and 10,000 ppm. For *C. gestroi*, both untreated and treated sections at all concentrations were fully explored after seven days. However, the feeding activity discontinued after day-3. No survivor was found in sand treated with 5,000 ppm and 10,000 ppm of *P. niruri*. The tunneling and penetrating activities was also unlikely after seven days of exposure. While other plant extracts caused 0% survivorship only in sand treated with 10,000 ppm.

In hexane extracts, *C. gestroi* was found to forage freely between two sections along the exposure period compared to *G. sulphureus*. *C. gestroi* showed significant effects of tunneling activity, *X*^2^(3) = 8.215, *p* = 0.042, with a mean rank treated section score of 32.92 for *P. niruri*, 24.58 for *A. paniculata*, 21.67 for *A. indica* and 18.83 for *L. leucocephala* (Table 3). A Kruskal-Wallis H test showed that there was no significant effect on tunneling activity of *G. sulphureus* in sand treated with different plant extracts (*X*^2^(3) = 5.831, *p* = 0.120) (Table 4). Low tunneling activity was observed in the treated section compared to untreated section. The feeding activity of both termites on treated and untreated sections generally ceased after three days. Hexane extract of *P. niruri* and *A. indica* caused 0% of tunneling activity while low tunneling activity was observed in *A. paniculata* and *L. leucocephala*. Hexane extracts of *P. niruri* caused a high mortality in both termites especially at a concentration of 10,000 ppm.

Sand treated with water extracts had the highest number of survivors compared to hexane and methanolic extracts (Table 5 and Table 6). From Kruskal-Walis H analysis, *C. gestroi* showed no significant effect of tunneling activity against different sand treated with plant extracts, *X*^2^(3) = 4.779, *p* = 0.189, whereas *G. sulphureus* displayed significant different of tunneling activity against different treated sands, *X*^2^(3) = 25.573, *p* ≤ 0.0001, with a mean rank of 38.67 for *P. niruri*, 17.00 for *A. paniculata*, 20.33 for *A. indica* and 22.00 for *L. leucecephala*. In the bioassay arena, termites were equally distributed throughout the arena and were present in both treated and untreated sections. Both termites were able to walk during seven days of exposure without any visible side effect from water extracts. Furthermore, both termites were able to tunnel in all untreated sections. Water extracts was observed to have no repellent insecticidal properties as all the treatments were completely foraged by *C. gestroi*. Although the mortality of *G. sulphureus* was observed in water extracts of *A. indica* and *A. paniculata*, however, the percentage of mortality was low.

### Effects of Plant Extracts on Feeding Activity of *C. gestroi* and *G. sulphureus*

A univariate analysis was conducted to examine the effect of solvent, plant and concentration on feeding activity of *C. gestroi* and *G. sulphureus* on treated sections. There was a statistically significant interaction between the effects of solvent, plant and concentration on feeding activity of *C. gestroi* (F (18, 96) = 1.719, *p* = 0.049). They consumed all the filter papers on both treated and untreated sections with hexane and water plant extracts (Table 3 and Table 6). In addition, all filter papers on sand treated with methanolic plant extracts were consumed by *C. gestroi* except for *P. niruri* at 5,000 and 10,000 ppm.

There was no significant difference between the effects of solvent, plant and concentration on the feeding consumption of *G. sulphureus*, F (18.96) = 1.282, *p* = 0.217. In methanolic plant extracts, the filter paper in the treated section was not consumed, except in the control section (Table 1). Meanwhile in hexane extracts, only *P. niruri* and *A. indica* caused zero consumption in the treated section. In water extracts, all filter papers were fully consumed by termites except for *A. indica* at 10,000 ppm.

### Behavioural Responses of *C. gestroi* and *G. sulphureus*

Based on the results, the termite behaviours were influenced by plant extracted with different solvents where different behaviours of *C. gestroi* and *G. sulphureus* were noted after seven days of exposure (Table 7). Both termites showed the lowest degree of tunneling activity when treated with methanolic extracts compared to hexane and water extracts. The consumption of filter paper on sand treated with methanolic and hexane plant extracts was slowly reduced over time exposure although some termites were alive in certain treatments, indicating that no active feeding activity occurred. Termites treated with methanolic and hexane plant extracts showed avoidance behaviour towards the treated sand, slowly losing the ability to walk and preferably stayed on untreated sand. Necrophobic behaviour was only present on the beginning of the exposure and they abandoned the dead termites after two days. Meanwhile, water extract treatment had low negative effects on *C. gestroi* and *G. sulpherus*. Moreover, they showed no avoidance from a water-treated section and move freely between both sections.

## DISCUSSION

This study demonstrated the potential use of crude solvent extracts of *A. paniculata, L. leucocephala*, *A. indica* and *P. niruri* as biocontrol agents against two termite species; *C. gestroi* and *G. sulphureus*. According to Yeoh and Lee (2007), termiticides used is required to be slow-acting and non-repellent for colony suppression and elimination due to termite necrophobic behaviour. Su *et al*. (1987) defined slow-acting insecticides as those killing 90% of the treated individuals within 14 days, producing a broad effective lethal-time 90%. The non-repellency and delayed action may impact the subterranean termite populations (Su 2005). Su (2005) added that the quick killing of the poisoned termites signals the healthy termites to simultaneously seal or abandon the tunnels leading to the treated zones. These slow-acting and non-repellency termiticides such as chlorpyrifos and chlordane typically kill termites by contact (Rust & Saran 2006). In a previous study by Thorne and Breisch (2001), the tested termites did not show any avoidance behaviour when exposed to imidacloprid, a non-repellent termiticide. The termites continued penetrating the treated soil, which eventually resulting in the colony suppression. Thus, when slow-acting insecticides are used, the level of mortality and the speed of killing depend on the insecticide concentration (Su *et al*. 1987).

In this study, methanolic plant extracts showed a strong repellent activity against *C. gestroi* and *G. sulphureus*. Meanwhile, hexane extracts showed repellent activity against *G. sulphureus* only. The repellent activity, however, was not observed in water extracts. Due to non-repellent activity in water extracts, the survivorship of both termites was high even after seven days of exposure. Among the methanolic extracts of *P. niruri* was found to be highly repellent and toxic where high mortality was noted among members of *C. gestroi* and *G. sulphureus*. The workers also consumed fewer filter papers in the treated sections than in controls. *P. niruri* is a rich source of phytochemicals such as alkaloids, carboxylic acids, terpenes, tannins, saponins and flavonoids such as quercetin, quercetol, quercetrin and rutin (Mellinger *et al*. 2005; Ambali *et al*. 2010). Some of these compounds are toxic to pests. Alkaloids are known to have insecticidal and fungicidal properties (Chowński *et al*. 2016), while, flavonoids protect the plant against insect pests by influencing the behaviour, growth and development of insects in plant defense mechanisms (Simmonds 2003). In the previous study, leaf extract from *P. niruri* was found to be effective in controlling *Aedes aegypti* at lower doses compared to marketed synthetic insecticides (Suresh *et al*. 2015). In laboratory assay, *P. niruri* was toxic to larval instar and pupae of *A. aegypti*. The number of larvae was also decreased in the field application. In addition, Umarani (2009) proved that leaf extract of *P. niruri* was toxic to *Culex quinquefasciatus* larvae, causing 100% mortality at 1,000 ppm. An extract of *P. amarus* which belongs to the same genus with *P. niruri* showed the mortality effect on *Macrotermes bellicosus* through topical application.

In this study, the tunneling activity and survivorship of both termites were dependent on the species and concentration of the extracts. In tunneling bioassay, termites slowly became immobile and their body sizes greatly decreased before they died. Tunneling behaviour and reduced capacity to walk reflected health declination among intoxicated termites (Strack & Myles 1997). Tunneling and walking ability of intoxicated termites are two important factors affecting their potential to transfer toxicants to other nestmates (Quarcoo *et al*. 2012). Although the feeding activity has stopped in treated sections, trophollaxis and grooming activities continuously occurred in untreated sections. According to Quarcoo *et al*. (2012), termites that lost their ability in tunneling and walking would likely reduce horizontal transfer to other colony members. In this study, although methanolic extract of *P. niruri* had caused termites to tunnel in untreated sections and no activity was observed in the treated zones, termites were killed in this treatment. A similar observation was reported in previous studies, of which, pyrethroid (tralomethrin) traveled from a treated sand to an agar layer resulted in high mortality in *Reticulitermes flavipes* even though the termite did not reach the treated area (Su & Scheffrahn 1990). A treatment using Bifentrin on *C. gestroi* also exhibited a similar result (Yeoh & Lee 2007). Raina *et al*. (2007) suggested that a vapour action might involve in termite mortality. The vapour from filter papers treated with orange oil extract moved to the untreated section containing *C. formosanus* which subsequently caused high mortality in this termite species. A previous report by Sharma *et al*. (1994) proved that the application of eucalyptus oil at 5 mg/ml^2^ caused 100% mortality of *Odontotermes bruneus*. The vapour phase of chlorinated hydrocarbons affecting the termite species has been reported by Ebeling and Pence (1958) where these compounds were able to penetrate into areas beyond the treated soil and eventually killed the termites. Yeoh and Lee (2007) postulated that termites might have a right amount of toxicant deposited on their cuticle when the vapour pressure is high enough. A penetration of toxicant through the cuticle will slowly affect the termites without direct contact (Su *et al*. 1982). Therefore, it can be assumed that a vapor action might also involve in affecting the behaviour of the termites tested in this study.

## CONCLUSION

In summary, all methanolic plant extracts displayed high repellent activity against *C. gestroi* and *G. sulphureus* compared to water and hexane extracts. Among extracted plants, *P. niruri* exhibited a good toxicity and repellency against both termite species. The present results also demonstrated that the mortality of termites was attributed to the duration of exposure and concentrations of the extracts. Further investigations are thus needed to assess any active compound present in methanolic plant extracts and their mode of action against higher and lower termites.

## Figures and Tables

**Table 1:** Performance of methanol extracts against *G. sulphureus* using Petri dish method.

Termite species	Solvent	Plant	Concentration (ppm)	Termite activity in treated section^1^	Filter paper consumption in treated section^2^	Termite activity in untreated section^1^	Filter paper consumption in untreated section^2^	Termite survivorship^2^
*G. sulphureus*	Methanol	*P. niruri*	10,000	0.000 ± 0.000a	0.000 ± 0.000a	0.667 ± 0.333a	0.001 ± 0.000a	0.000 ± 0.000a
			5,000	0.000 ± 0.000a	0.000 ± 0.000a	0.333 ± 0.333a	0.001 ± 0.001a	0.000 ± 0.000a
			500	0.000 ± 0.000a	0.000 ± 0.000a	0.333 ± 0.333a	0.000 ± 0.000a	0.000 ± 0.000a
			Control	1.000 ±0.000a	0.003 ± 0.001a	1.000 ± 0.000a	0.003 ± 0.000b	100.000 ± 0.000b
		*A. paniculata*	10,000	0.000± 0.000a	0.000 ± 0.000a	0.000 ± 0.000a	0.001 ± 0.000a	0.000 ± 0.000a
			5,000	0.000 ± 0.000a	0.000 ± 0.000a	0.000 ± 0.000a	0.002 ± 0.001ab	0.000 ± 0.000a
			500	0.000 ± 0.000a	0.000 ± 0.000a	0.000 ± 0.000a	0.002 ± 0.001ab	19.231 ± 19.23a
			Control	0.667 ± 0.333a	0.002 ± 0.001a	0.000 ± 0.000a	0.002 ± 0.001ab	91.026 ± 4.203b
		*A. indica*	10,000	0.000 ± 0.000a	0.000 ± 0.000a	0.000 ± 0.000a	0.001 ± 0.000a	0.000 ± 0.000a
			5,000	0.000 ± 0.000a	0.000 ± 0.000a	0.000 ± 0.000a	0.001 ± 0.000a	3.846 ± 1.923a
			500	0.000 ± 0.000a	0.000 ± 0.000a	0.000 ± 0.000a	0.002 ± 0.001ab	0.000 ± 0.000a
			Control	0.667 ± 0.333a	0.002 ± 0.001a	0.333 ± 0.333a	0.002 ± 0.001ab	85.256 ± 1.696b
		*L. leucocephala*	10,000	0.000 ± 0.000a	0.000 ± 0.000a	0.000 ± 0.000a	0.003 ± 0.000b	0.000 ± 0.000a
			5,000	0.000 ± 0.000a	0.000 ± 0.000a	0.000 ± 0.000a	0.002 ± 0.001ab	0.000 ± 0.000a
			500	0.000 ± 0.000a	0.000 ± 0.000a	0.000 ± 0.000a	0.004 ± 0.001b	11.539 ± 5.875a
			Control	0.667 ± 0.333a	0.003 ± 0.000a	1.000 ± 0.000a	0.002 ± 0.001ab	86.539 ± 1.110b

Notes:

1 Intensity of tunnels formed: 0 = no tunneling activity; 1 = tunneling activities covering ≤ 25% of total arena; 2 = tunneling activities covering 26–50% of total arena; 3 = tunneling activities covering 51–75% of total arena; 4 = tunneling activities covering ≥ 75% of total arena.

Means followed by the different letters within the same column are significantly different (Kruskal-Wallis multiple Range Test; P < 0.05).

2 Means followed by different letters within the same column are significantly different (Tukey HSD; P < 0.05).

Value are mean ± s.e

**Table 2:** Performance of methanol extracts against *C. gestroi* using Petri dish method.

Termite species	Solvent	Plant	Concentration (ppm)	Termite activity in treated section^1^	Filter paper consumption in treated section^2^	Termite activity in untreated section^1^	Filter paper consumption in untreated section^2^	Termite survivorship^2^
C. gestroi	Methanol	*P. niruri*	10,000	0.000 ± 0.000a	0.000 ± 0.000a	1.000 ± 0.000a	0.002 ± 0.001a	0.000 ± 0.000aa
			5,000	0.000 ± 0.000a	0.000 ± 0.000a	1.000 ± 0.000a	0.001 ± 0.000a	0.000 ± 0.000a
			500	1.000 ± 0.000a	0.000 ± 0.000a	1.000 ± 0.000a	0.002 ± 0.001a	5.769 ± 2.938a
			Control	1.000 ± 0.000a	0.003 ± 0.001b	1.000 ± 0.000a	0.003 ± 0.000a	98.077 ± 1.923b
		*A. paniculata*	10,000	0.333 ± 0.333a	0.002 ± 0.001b	1.000 ± 0.000a	0.002 ± 0.001a	0.000 ± 0.000a
			5,000	0.667 ± 0.333a	0.002 ± 0.001b	1.000 ± 0.000a	0.003 ± 0.001a	10.256 ± 3.392a
			500	1.000 ± 0.000a	0.004 ± 0.000b	1.000 ± 0.000a	0.003 ± 0.001a	50.641 ± 22.048ab
			Control	1.000 ± 0.000a	0.003 ± 0.001b	1.000 ± 0.000a	0.003 ± 0.000a	94.231 ± 2.938b
		*A. indica*	10,000	0.667 ± 0.333a	0.001 ± 0.000a	1.000 ± 0.000a	0.002 ± 0.000a	0.000 ± 0.000a
			5,000	0.667 ± 0.333a	0.001 ± 0.001a	0.667 ± 0.333a	0.002 ± 0.000a	2.564 ± 2.564a
			500	0.667 ± 0.333a	0.002 ± 0.001b	1.000 ± 0.000a	0.002 ± 0.001a	10.897 ± 6.313ab
			Control	1.000 ± 0.000a	0.002 ± 0.001b	1.000 ± 0.000a	0.002 ± 0.001a	98.077 ± 1.110b
		*L. leucocephala*	10,000	0.667 ± 0.333a	0.003 ± 0.000b	1.000 ± 0.000a	0.002 ± 0.001a	0.000 ± 0.000a
			5,000	1.000 ± 0.000a	0.002 ± 0.001b	1.000 ± 0.000a	0.002 ± 0.001a	0.000 ± 0.000a
			500	1.333 ± 0.333a	0.003 ± 0.001b	1.000 ± 0.000a	0.004 ± 0.001a	26.282 ± 13.793ab
			Control	1.333 ± 0.333a	0.002 ± 0.001b	1.333 ± 0.333a	0.003 ± 0.001a	98.077 ± 1.923b

Notes:

1 Intensity of tunnels formed: 0 = no tunneling activity; 1 = tunneling activities covering ≤ 25% of total arena; 2 = tunneling activities covering 26–50% of total arena; 3 = tunneling activities covering 51–75% of total arena; 4 = tunneling activities covering ≥ 75% of total arena.

Means followed by the different letters within the same column are significantly different (Kruskal-Wallis multiple Range Test; P < 0.05).

2 Means followed by different letters within the same column are significantly different (Tukey HSD; P < 0.05).

Value are mean ± s.e

**Table 3:** Performance of hexane extracts against *C. gestroi* using Petri dish method.

Termite species	Solvent	Plant	Concentration (ppm)	Termite activity in treated section^1^	Filter paper consumption in treated section 2	Termite activity in untreated section^1^	Filter paper consumption in untreated section 2	Termite survivorship^2^
*C. gestroi*	Hexane	*P. niruri*	10,000	3.000 ± 1.000a	0.001 ± 0.001a	2.000 ± 0.000a	0.002 ± 0.001a	37.821 ± 6.313a
			5,000	3.667 ± 0.333a	0.002 ± 0.000a	2.000 ± 0.000a	0.002 ± 0.001a	58.974 ± 8.407a
			500	2.000 ± 0.577a	0.002 ± 0.000a	2.000 ± 0.577a	0.002 ± 0.000a	59.615 ± 4.840a
			Control	2.667 ± 0.882a	0.001 ± 0.000a	1.000 ± 0.000a	0.001 ± 0.000a	95.513 ± 0.641ab
		*A. paniculata*	10,000	2.667 ± 0.667a	0.001 ± 0.000a	1.667 ± 0.333a	0.002 ± 0.000a	71.795 ± 7.798a
			5,000	1.667 ± 0.333a	0.001 ± 0.000a	1.333 ± 0.333a	0.001 ± 0.000a	74.359 ± 4.623a
			500	2.333 ± 0.667a	0.002 ± 0.001a	1.000 ± 0.000a	0.003 ± 0.001a	67.949 ± 2.311a
			Control	2.000 ± 0.000a	0.002 ± 0.001a	1.000 ±0.000a	0.001 ± 0.001a	91.667 ± 1.696ab
		*A. indica*	10,000	2.333 ± 0.333a	0.001 ± 0.000a	2.667 ± 0.667a	0.000 ± 0.000a	62.821 ± 3.205a
			5,000	1.667 ± 0.333a	0.001 ± 0.000a	1.333 ± 0.333a	0.001 ± 0.000a	60.897 ± 7.558a
			500	1.667 ± 0.333a	0.001 ± 0.000a	2.000 ± 0.577a	0.001 ± 0.001a	65.385 ± 6.662a
			Control	2.333 ± 0.333a	0.002 ± 0.001a	1.333 ± 0.333a	0.002 ± 0.000a	89.103 ± 1.696ab
		*L. leucocephala*	10,000	3.000 ± 0.000a	0.001 ± 0.000a	2.000 ± 0.000a	0.001 ± 0.000a	53.846 ± 10.591a
			5,000	2.667 ± 0.333a	0.001 ± 0.000a	1.333 ± 0.333a	0.002 ± 0.000a	48.077 ± 4.003a
			500	2.000 ± 0.000a	0.001 ± 0.000a	1.000 ± 0.000a	0.001 ± 0.000a	76.282 ± 0.641a
			Control	1.667 ± 0.333a	0.001 ± 0.001a	1.000 ± 0.000a	0.001 ± 0.000a	85.897 ± 3.392ab

Notes:

1 Intensity of tunnels formed: 0 = no tunneling activity; 1 = tunneling activities covering ≤ 25% of total arena; 2 = tunneling activities covering 26–50% of total arena; 3 = tunneling activities covering 51–75% of total arena; 4 = tunneling activities covering ≥ 75% of total arena.

Means followed by the different letters within the same column are significantly different (Kruskal-Wallis multiple Range Test; P < 0.05).

2 Means followed by different letters within the same column are significantly different (Tukey HSD; P < 0.05).

Value are mean ± s.e

**Table 4:** Performance of hexane extracts against *G. sulphureus* using Petri dish method.

Termite species	Solvent	Plant	Concentration (ppm)	Termite activity in treated section^1^	Filter paper consumption in treated section^2^	Termite activity in untreated section^1^	Filter paper consumption in untreated section^2^	Termite survivorship^2^
*G. sulphureus*	Hexane	*P. niruri*	10,000	0.000 ± 0.000a	0.000 ± 0.000a	1.0 ± 0.0b	0.001 ± 0.000a	19.872 ± 2.311a
			5,000	0.000 ± 0.000a	0.000 ± 0.000a	1.0 ± 0.0b	0.001 ± 0.000a	31.410 ± 5.698a
			500	0.000 ± 0.000a	0.000 ± 0.000a	1.0 ± 0.0b	0.001 ± 0.000a	37.821 ± 1.696a
			Control	1.000 ± 0.000b	0.001 ± 0.000a	1.0 ± 0.0b	0.002 ± 0.000ab	87.180 ± 2.794b
		*A. paniculata*	10,000	0.333 ± 0.333ab	0.000 ± 0.000a	1.0 ± 0.0b	0.002 ± 0.000ab	24.359 ± 0.641a
			5,000	0.000 ± 0.000a	0.001 ± 0.000a	1.0 ± 0.0b	0.001 ± 0.000a	40.385 ± 4.003ab
			500	0.333 ± 0.333ab	0.000 ± 0.000a	1.0 ± 0.0b	0.003 ± 0.000b	58.333 ± 7.138ab
			Control	1.000 ± 0.000b	0.002 ± 0.001ab	0.0 ± 0.0a	0.001 ± 0.000a	90.385 ± 2.938b
		*A. indica*	10,000	0.000 ± 0.000a	0.000 ± 0.000a	1.0 ± 0.0b	0.000 ± 0.000a	44.872 ± 0.641ab
			5,000	0.000 ± 0.000a	0.000 ± 0.000a	1.0 ± 0.0b	0.001 ± 0.000a	51.923 ± 4.840ab
			500	0.000 ± 0.000a	0.000 ± 0.000a	1.0 ± 0.0b	0.001 ± 0.001a	48.718 ± 1.696ab
			Control	0.667 ± 0.333ab	0.002 ± 0.000ab	0.0 ± 0.0a	0.001 ± 0.000a	91.026 ± 0.641b
		*L. leucocephala*	10,000	0.000 ± 0.000a	0.000 ± 0.000a	1.0 ± 0.0b	0.000 ± 0.000a	46.795 ± 1.696ab
			5,000	1.000 ± 0.000b	0.001 ± 0.000a	0.0 ± 0.0a	0.001 ± 0.000a	49.359 ± 0.641ab
			500	0.667 ± 0.333ab	0.001 ± 0.000a	1.0 ± 0.0b	0.001 ± 0.000a	47.436 ± 1.282ab
			Control	0.667 ± 0.333ab	0.003 ± 0.000ab	0.0 ± 0.0a	0.002 ± 0.000ab	91.667 ± 1.282b

Notes:

1 Intensity of tunnels formed: 0 = no tunneling activity; 1 = tunneling activities covering ≤ 25% of total arena; 2 = tunneling activities covering 26–50% of total arena; 3 = tunneling activities covering 51–75% of total arena; 4 = tunneling activities covering ≥ 75% of total arena.

Means followed by the different letters within the same column are significantly different (Kruskal-Wallis multiple Range Test; P < 0.05).

2 Means followed by different letters within the same column are significantly different (Tukey HSD; P < 0.05).

Value are mean ± s.e

**Table 5:** Performance of water extracts against *G. sulphureus* using petri dish method.

Termite species	Solvent	Plant	Concentration (ppm)	Termite activity in treated section^1^	Filter paper consumption in treated section 2	Termite activity in untreated section^1^	Filter paper consumption in untreated section 2	Termite survivorship^2^
*G. sulphureus*	Water	*P. niruri*	10,000	3.000 ± 1.732a	0.002 ± 0.001a	2.000 ± 0.000a	0.002 ± 0.001a	96.154 ± 0.000a
			5,000	3.667 ± 0.577a	0.002 ± 0.000a	1.333 ± 0.333a	0.002 ± 0.001a	97.436 ± 0.641a
			500	2.000 ± 1.000a	0.002 ± 0.000a	2.000 ± 0.577a	0.002 ± 0.000a	96.154 ± 1.110a
			Control	0.333 ± 0.577a	0.003 ± 0.001a	0.333 ± 0.333a	0.002 ± 0.000a	99.359 ± 0.641a
		*A. paniculata*	10,000	0.000 ± 0.000a	0.000 ± 0.000a	0.333 ± 0.333a	0.001 ± 0.000a	99.359 ± 0.641a
			5,000	0.000 ± 0.000a	0.001 ± 0.000a	0.667 ± 0.333a	0.002 ± 0.000a	98.718 ± 0.641a
			500	0.000 ± 0.000a	0.000 ± 0.000a	0.333 ± 0.333a	0.002 ± 0.001a	99.359 ± 0.641a
			Control	0.000 ± 0.000a	0.001 ± 0.000a	0.333 ± 0.333a	0.002 ± 0.000a	99.359 ± 0.641a
		*A. indica*	10,000	0.000 ± 0.000a	0.000 ± 0.000a	1.000 ± 0.000a	0.003 ± 0.000a	98.077 ± 0.000a
			5,000	0.000 ± 0.000a	0.001 ± 0.000a	1.000 ± 0.000a	0.002 ± 0.001a	98.077 ± 0.000a
			500	0.000 ± 0.000a	0.001 ± 0.000a	0.000 ± 0.000a	0.002 ± 0.000a	100.000 ± 0.000a
			Control	0.667 ± 0.577a	0.002 ± 0.000a	0.667 ± 0.333a	0.002 ± 0.001a	98.718 ± 0.641a
		*L. leucocephala*	10,000	0.333 ± 0.577a	0.002 ± 0.000a	0.333 ± 0.333a	0.002 ± 0.001a	99.359 ± 0.641a
			5,000	0.333 ± 0.577a	0.001 ± 0.001a	0.333 ± 0.333a	0.002 ± 0.000a	99.359 ± 0.641a
			500	0.000 ± 0.000a	0.003 ± 0.001a	0.667 ± 0.333a	0.002 ± 0.001a	98.718 ± 0.641a
			Control	0.333 ± 0.577a	0.001 ± 0.001a	0.000 ± 0.000a	0.001 ± 0.000a	100.000 ± 0.000a

Notes:

1 Intensity of tunnels formed: 0 = no tunneling activity; 1 = tunneling activities covering ≤ 25% of total arena; 2 = tunneling activities covering 26–50% of total arena; 3 = tunneling activities covering 51–75% of total arena; 4 = tunneling activities covering ≥ 75% of total arena.

Means followed by the different letters within the same column are significantly different (Kruskal-Wallis multiple Range Test; P < 0.05).

2 Means followed by different letters within the same column are significantly different (Tukey HSD; P < 0.05).

Value are mean±s.e

**Table 6:** Performance of water extracts against C. gestroi using petri dish method.

Termite species	Solvent	Plant	Concentration (ppm)	Termite activity in treated section^1^	Filter paper consumption in treated section 2	Termite activity in untreated section^1^	Filter paper consumption in untreated section 2	Termite survivorship^2^
*C. gestroi*	Water	*P. niruri*	10000	3.000 ± 1.000a	0.003 ± 0.001a	2.000 ± 0.000a	0.003 ± 0.001a	96.154 ± 2.938a
			5000	3.667 ± 0.333a	0.002 ± 0.000a	1.667 ± 0.333a	0.003 ± 0.001a	99.359 ± 0.641a
			500	2.000 ± 0.577a	0.002 ± 0.000a	2.333 ± 0.882a	0.002 ± 0.000a	88.462 ± 6.182a
			Control	2.667 ± 0.882a	0.002 ± 0.001a	3.333 ± 0.667a	0.003 ± 0.001a	100.000 ± 0.000a
		*A. paniculata*	10000	2.667 ± 0.667a	0.002 ± 0.001a	2.333 ± 0.333a	0.002 ± 0.000a	98.718 ± 0.641a
			5000	1.667 ± 0.333a	0.002 ± 0.000a	1.000 ± 0.000a	0.002 ± 0.001a	87.821 ± 9.311a
			500	2.333 ± 0.667a	0.003 ± 0.000a	1.333 ± 0.333a	0.003 ± 0.000a	97.436 ± 2.564a
			Control	2.000 ± 0.000a	0.003 ± 0.001a	2.333 ± 0.882a	0.002 ± 0.001a	100.000 ± 0.000a
		*A. indica*	10000	2.333 ± 0.333a	0.001 ± 0.000a	1.333 ± 0.333a	0.002 ± 0.001a	99.359 ± 0.641a
			5000	1.667 ± 0.333a	0.001 ± 0.000a	2.000 ± 0.000a	0.001 ± 0.000a	95.513 ± 4.487a
			500	1.667 ± 0.333a	0.001 ± 0.000a	2.333 ± 0.333a	0.002 ± 0.001a	99.359 ± 0.641a
			Control	2.333 ± 0.333a	0.002 ± 0.000a	2.333 ± 0.667a	0.003 ± 0.001a	100.000 ± 0.000a
		*L. leucocephala*	10000	3.000 ± 0.000a	0.002 ± 0.000a	1.333 ± 0.333a	0.003 ± 0.001a	98.718 ± 1.282a
			5000	2.667 ± 0.333a	0.002 ± 0.001a	2.333 ± 0.667a	0.003 ± 0.000a	98.077 ± 1.110a
			500	2.000 ± 0.000a	0.003 ± 0.001a	1.667 ± 0.333a	0.001 ± 0.000a	98.077 ± 1.110a
			Control	1.667 ± 0.333a	0.003 ± 0.001a	2.000 ± 0.577a	0.002 ± 0.001a	100.000 ± 0.000a

Notes:

1 Intensity of tunnels formed: 0 = no tunneling activity; 1 = tunneling activities covering ≤ 25% of total arena; 2 = tunneling activities covering 26–50% of total arena; 3 = tunneling activities covering 51–75% of total arena; 4 = tunneling activities covering ≥ 75% of total arena.

Means followed by the different letters within the same column are significantly different (Kruskal-Wallis multiple Range Test; P < 0.05).

2 Means followed by different letters within the same column are significantly different (Tukey HSD; P < 0.05).

Value are mean ± s.e

**Table 7:** The comparison of different behaviour aspects for *G. sulphureus* and *C. gestroi*.

Treatment	Behaviour aspects	Termite species	

		*G. sulphureus*	*C. gestroi*
Plant extracted with methanol	Avoidance on treated sand	Yes	Yes
	Grouping on untreated sand	Yes, as group	Yes, as group
	Movement	Not moving and active	Not moving and active
	Feeding	Stop after 2 days	Stop after 2 days
	Necrophobic	No	No

Plant extracted with hexane	Avoidance on treated sand	Yes	Only on certain plant
	Grouping on untreated sand	Yes	Only on certain plant
	Movement	Slow and not active	Slow and not active
	Feeding	Stop after 3 days	Stop after 3 days
	Necrophobic	No	No

Plant extracted with water	Avoidance on treated sand	No	No
	Grouping on untreated sand	No	No
	Movement	Very active, fast and move freely between both sides	Very active, fast and move freely between both sides
	Feeding	Slowly reduced over time	Slowly reduced over time
	Necrophobic	Frequent	Frequent
